# Elimination of Arctic Variant Rabies in Red Foxes, Metropolitan Toronto

**DOI:** 10.3201/eid1301.060622

**Published:** 2007-01

**Authors:** R. C. Rosatte, M. J. Power, D. Donovan, J. C. Davies, M. Allan, P. Bachmann, B. Stevenson, A. Wandeler, F. Muldoon

**Affiliations:** * Ontario Ministry of Natural Resources, Peterborough, Ontario, Canada; † Ontario Ministry of Natural Resources, Maple, Ontario, Canada; ‡ Canadian Food Inspection Agency, Nepean, Ontario, Canada; 1 Now retired.

**Keywords:** Ontario, rabies, red fox, Vulpes vulpes, research

## Abstract

To control the arctic variant of rabies virus in red foxes, 332,257 bait doses containing live, attenuated Evelyn-Rokitnicki-Abelseth rabies vaccine were distributed in greater metropolitan Toronto during 1989–1999. Human and pet contact with bait was minimal, and no adverse reactions to the vaccine were noted. Significantly fewer rabid foxes were found during the 17 years after fox baiting (5 cases during 1990–2006) than in the 17 years before (96 cases during 1973–1989). The last report of a rabid fox in metropolitan Toronto was in 1996 (reporting period through September 2006), which confirms that distributing oral rabies vaccine bait is a feasible tactic for the control of rabies in foxes in urban environments.

The arctic variant of rabies virus has been present in red fox (*Vulpes vulpes*) populations in Ontario, Canada, since the mid-1950s (*1*,*2*). During 1954–2006, more than 57,000 rabid animals were reported in Ontario, and, on average, 1,000–2,000 humans received rabies postexposure treatment (*3*,*4*). Before rabies control programs were implemented, red foxes accounted for ≈45% of all rabies cases in Ontario (*2*,*5*). In metropolitan Toronto, rabies was cyclic from the 1960s to the 1980s; outbreaks in red foxes and striped skunks (*Mephitis mephitis)* occurred every 2 to 5 years (Figure 1).

**Figure 1:**
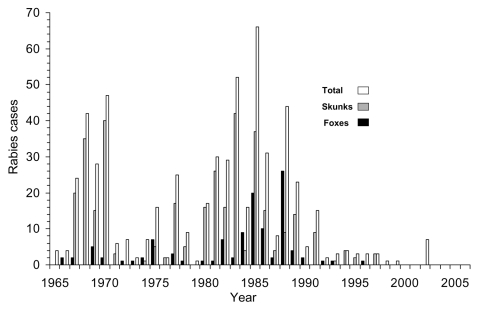
Rabies cases in metropolitan Toronto, 1965–2006. Total includes all species that were reported rabid, most of which were bats.

## Methods

**Figure 2:**
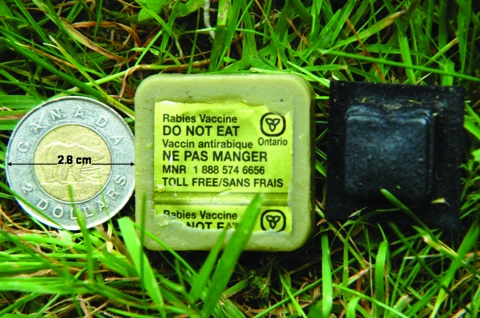
Evelyn-Rokitnicki-Abelseth rabies vaccine bait, showing the vaccine container (normally embedded in the matrix of the bait) to the right of the bait. Photograph, Ontario Ministry of Natural Resources Rabies Unit.

Beginning in 1989, oral vaccination using bait that contained the live, attenuated, Evelyn-Rokitnicki-Abelseth (ERA) (*6*,*7*) strain of rabies virus was distributed in Ontario to control rabies in red foxes in rural and urban habitats (*4*,*8*–*10*). The bait matrix consisted of beef tallow, wax, and attractants such as chicken or cod (*6*). The vaccine was contained in a blister pack, which was embedded in the matrix of the bait (Figure 2). Vaccine-bait components are described in more detail by Bachmann et al. (*6*) and Rosatte et al. (*9*) (Figure 2).

**Figure 3:**
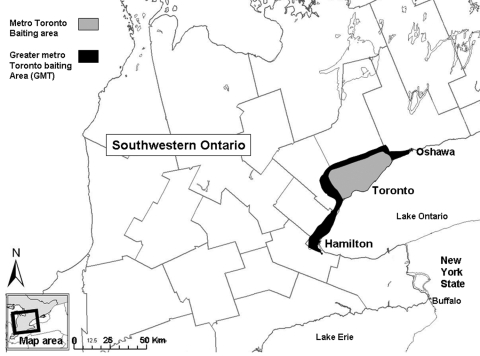
Greater metropolitan Toronto area where rabies vaccine bait doses were distributed during 1989–1999.

The metropolitan Toronto area (centered at latitude 43° 42′ north, longitude 79° 25′ west) was defined as a 638-km^2^ urban complex that included the cities of Toronto, North York, Etobicoke, East York, York, and North York. The program in metropolitan Toronto was expanded during 1994–1999 (Figure 3) to include the urban corridor from Oshawa to Hamilton (greater metropolitan Toronto, 1,850 km^2^) (Figure 3). During 1998–1999, baiting in rural southwestern Ontario extended into the greater metropolitan Toronto area (Figure 3). During the 1990s, ≈1,000 foxes (about 1.5/km^2^) lived in close proximity to ≈3 million people in metropolitan Toronto (*10*,*11*). In addition, during 1987–1996, trap-vaccinate-release programs (vaccination by hand-delivered injection) to control rabies in striped skunks and raccoons (*Procyon lotor*) were conducted in a 60-km^2^ portion of metropolitan Toronto (Scarborough) (*9*). From a public health perspective, rabies control was crucial because since the 1950s, >63,000 humans had been treated for exposure to potentially rabid animals in Ontario (*10*,*12*).

During 1989–1999, a total of 332,257 bait doses containing ERA rabies vaccine were distributed in Toronto and the greater metropolitan Toronto area (Table). Bait density was ≈49–69 doses/linear km of ravine (*10*). Bait was distributed primarily by personnel who walked (ground baiting) throughout the ravines and green-belt areas of greater metropolitan Toronto; ecologic studies of red foxes indicated that ravines are used as travel corridors by foxes (*11*). In 1994, 5,500 bait doses were also distributed aerially along the ravine systems from a Turbo Beaver aircraft flying at an altitude of 150 m with an approximate airspeed of 140 km/hr. In addition, during 1998, about 16,000 doses were aerially distributed in the greater metropolitan Toronto area by Twin-Otter aircraft. Timing of bait placement varied each year but was generally in June and November, from 1989 through 1999. Hand-baiting personnel tried to place 1 vaccine-bait dose every 50 m on both sides of waterways in the ravine systems (*9*). As a modified live virus rabies vaccine was being used in an urban setting, news releases were issued to the media before and during annual baiting operations. The primary objective of the media campaign was to notify the public of the program and ask people not to touch the bait.

**Table:** Number of rabies vaccine bait doses distributed in metropolitan and greater metropolitan Toronto*

Year	Region	Ground placement	Aerial distribution
1989	Metropolitan Toronto	10,262	
1990	Metropolitan Toronto	27,535	
1991	Metropolitan Toronto	28,371	
1992	Metropolitan Toronto	21,635	
1993	Metropolitan Toronto	24,992	
1994	GMT	40,000	6,000
1995	GMT	45,509	
1996	GMT	41,000	
1997	GMT	40,000	
1998	GMT	15,953	16,000
1999	GMT	15,000	

*Total no. bait doses distributed, 332,257; GMT, greater metropolitan Toronto.

## Results

We documented that 15 persons found bait (but did not touch the vaccine) and 22 dogs had contact with or consumed the bait during hand-baiting operations. Clinical signs in dogs after bait ingestion sometimes included diarrhea or vomiting (most likely attributable to the tallow and wax in the bait). Three of the dogs had intestinal problems, and 1 had an intestinal obstruction, likely caused by the blister pack. After the 1994 aerial baiting campaign, only 5 persons reported finding bait in their yards. The time needed to hand-distribute ≈28,000 vaccine-bait doses each year in metropolitan Toronto was ≈145 person-days, which is ≈193 bait doses/person/day. The annual cost to hand-distribute these ≈28,000 bait doses was about Can $25,000 for labor, travel expenses, vehicles, and gas plus ≈$30,000 for the bait (total cost of ≈$1.96/dose).

Acceptance of vaccine-bait was determined by the presence of tetracycline in tooth sections (6). Bait acceptance by foxes sampled in metropolitan Toronto during 1989–1991 was 55%–80%, and rabies antibody was detected in 74%–100% of the foxes that consumed the bait (*9*). During this period, 50%–68% of the foxes were vaccinated each year (*9*,*10*). Significantly fewer rabid foxes were reported in metropolitan Toronto during the 17 years after fox baiting began (1990–2006, 5 cases, mean 0.3/yr, standard deviation [SD] 0.6) than during the 17 years before baiting began (1973–1989, 96 cases, mean 5.7/yr, SD 7.3) (t=3.01, p<0.005) (Figure 1). On the basis of the cyclic nature of outbreaks in metropolitan Toronto of rabies in foxes (every 2–5 years), as well as in skunks, an outbreak should have occurred during the mid-1990s; but no outbreak occurred. As of September 2006, the last rabid fox in metropolitan Toronto, as well as the greater metropolitan Toronto area, had been reported in 1996. Distribution of vaccine-bait in that urban complex was discontinued in 2000 because metropolitan Toronto had been free from reported rabies in foxes for 3 years.

## Discussion

Metropolitan Toronto is connected to rural areas through a series of ravine systems dominated primarily by deciduous trees. These ravines provide a travel corridor through which wildlife, including red foxes, moves into and out of metropolitan Toronto (*11*). The ground and aerial distribution of rabies vaccine bait in metropolitan and greater metropolitan Toronto, which resulted in immunization of a substantial portion of the fox population against rabies, eliminated rabies from that urban complex. Aerial baiting in rural habitats surrounding metropolitan Toronto, as well as greater metropolitan Toronto, after 1995 may have contributed to rabies control in metropolitan Toronto, as few rabid foxes have been available to disperse rabies into that urban complex. As well, one cannot discount the effect that the trap-vaccinate-release programs in Scarborough had on the control of rabies in metropolitan Toronto. However, the trap-vaccinate-release program targeted raccoons and skunks as opposed to foxes (*9*). Greater metropolitan Toronto has been free of reported cases of rabies in red foxes for a decade (1997–2006) and is a notable success for the Ontario Ministry of Natural Resources rabies control programs. The results of this program confirm that distribution of oral rabies vaccine bait is a feasible tactic for controlling rabies in foxes in urban environments.
